# Macro-seepage based potential new hydrocarbon prospects in Assam-Arakan Basin, India

**DOI:** 10.1038/s41598-022-06045-6

**Published:** 2022-02-10

**Authors:** Annapurna Boruah, Sumit Verma, Abdul Rasheed, Gaurav Siddharth Gairola, Anuj Gogoi

**Affiliations:** 1 University of Petroleum and Energy Studies, Dehradun, India; 2 University of Texas Permian Basin, Odessa, USA; 3 Gujarat Energy Research and Management Institute, Gandhinagar, India; 4 King Abdullah University of Science and Technology, Thuwal, Saudi Arabia

**Keywords:** Crude oil, Natural gas, Geochemistry, Geology, Sedimentology, Tectonics, Microbiology, Energy science and technology, Energy

## Abstract

Active macro seepages of methane that occur in between the north bank of the Brahmaputra river and Himalayan foothill region of Assam Arakan Basin, India, indicate the presence of hydrocarbon accumulation in the subsurface, but the hydrocarbon prospects in this region are not well studied. We carried out an extensive field sampling, which included a total of 58 sediment core collections from an active gas seepage location and nearby areas at a depth of 2–2.5 m. Our sample locations are placed at 1 km intervals laterally. We performed laboratory investigations and mapped near-surface chemical alterations associated with active macro seepages and microseepages. The analysis of geochemical composition of hydrocarbon gases in the sediment indicates both the biogenic and thermogenic origins of seeped hydrocarbons. The stable isotope analysis of methane suggests the presence of thermogenic as well as mixed biogenic-thermogenic gases. The presence of such mixing of gases is caused by the secondary alteration processes during their migration through potential faults and fractures. The trace elements of the sediments show anomalous concentrations at different parts of the study area, with a wide range of concentrations for Ba (54 to 492 ppm), Cu (1–25 ppm), Cr (61–329 ppm), Ni (1–42 ppm), Pb (2–48 ppm), Th (2–32 ppm), U (4–39 ppm), V (19–133 ppm) and U (0.87–6.5 ppm). There are higher concentrations of adsorbed gases, trace elements, and microbes along the identified lineaments. Such higher concentration can be triggered by high hydrocarbon-oxidizing bacteria count, which is greater than 10^4^ cfu/gm of soil of bacterial growth around the lineaments. We identified potential hydrocarbon prospects based on the macro and micro seepage analysis using integrated geological, geochemical and microbial techniques in the study area.

## Introduction

Many of the proven commercial oil and gas fields, in both frontier and mature basins, were discovered based on oil or gas seepages^1–3^. A study on the global-scale distribution of geological methane emissions demonstrates that almost 81% of seeps occur within petroleum fields, and 19% of seeps occur due to the presence of minor petroleum pools or as a result of long-distance fluid migration from the source rocks^3^. The first petroleum seeps evidenced more than 40,000 years ago. Most of the initial petroleum wells were drilled in different parts of the world, based on seepages. For example, the first commercial oil well in Poland (1853), the first oil well in North America (1858), and the first commercial oil well in the USA on oil creek in Pennsylvania (1859) was drilled based on oil seepages^4^. Most of the petroleum discoveries in California during the 19th century were also based on observations of oil seeps. Moreover, until the actualization of seismic technology, surface seeps were considered the primary indicator of petroleum occurrences. The first petroleum discovery well in India was drilled in the Digboi oil field of Assam in 1889, based on the natural seepages of hydrocarbons^5^. The present study is focused on macro seepages occurring in the Gohpur region in Assam, where gas kicked at while drilling the water well and it has been flowing for more than 8 years. Such two active macro seepages and their nearby areas are considered for this study, and an attempt has been made to analyze the microseepage occurrences near those macro seepages in the study area.

Previous researchers classified hydrocarbon seepages into two categories: macroseepage and microseepage. Macroseepage are the large visible concentration of hydrocarbons on the surface, which can be considered as a direct hydrocarbon indicator (DHI) and the micro seepages are not visible, but can be noticed in the form of stains and odors. Seeped gases can migrate vertically as well as laterally via porous, permeable damage zones of faults, fractures or vents causing chemical changes in the soil^2,6–12^. Hydrocarbons flows within the reservoir rocks due to buoyancy force, while it can migrate from the source to the reservoir through capillary imbibition^13–15^. Generally, the seeped gases cause gas-soil interaction, elemental alteration or chemical changes in the strata overlying the hydrocarbon accumulation^16–19^. Surface geochemical exploration technique can be applied to analyze the surface or near-surface macro-seepages or microseepages of hydrocarbon, especially to identify the gaseous hydrocarbons that are adsorbed on the soil matrix in surface, or near-surface soils. Surface geochemical exploration is an unconventional but cost-effective technique to explore hydrocarbon prospects in the frontier areas as well as in mature oil fields. Measurements of trace elements concentration, microbial concentration in the soil sample, and adsorbed gas concentration are some of the common surface geochemical exploration techniques^1,10,13,16,17,20,21^. The trace elements anomalies in near-surface sediments above hydrocarbon reservoirs or source rocks are very common. Generally, these trace elements are found in higher concentrations around the periphery of hydrocarbon anomalies^8^.

Previous studies reported the trace elements as useful indicator for hydrocarbon microseepage identification^1,7,13,20,22^. Most of these studies correlated the trace elements concentrations with adsorbed soil gas results and demonstrated that how the trace elements concentrations vary near hydrocarbon anomalies. For example, the nickel (Ni) and vanadium (V) are the dominant trace elements present in oil and gas, V/Ni ratio has been used as an effective measure to determine hydrocarbon migration direction. V/Ni ratio decreases along the hydrocarbon migration direction, they can also appear as near-surface “haloes”^9,23^. Other than nickel (Ni) and vanadium (V), chromium (Cr), iron (Fe), cobalt (Co), copper (Co), manganese (Mn), strontium (Sr), barium (Ba) are also common in oil and gas. These trace elements can travel a long distance and occur as a halo. Trace element halos can be an indication of subsurface hydrocarbon accumulations^24^. Microseepage analysis carried out in different petroliferous regions of the Tatipaka and Pasarlapudi areas of Krishna Godavari Basin, India showed significantly higher concentrations of trace elements than normal concentrations in soils, i.e., V (197–489 mg/kg), Cr (106–287 mg/kg), Co (31–52 mg/kg), Ni (65–110 mg/kg), Cu (88–131 mg/kg), Zn (88–471 mg/kg), Ba (263–3091 mg/kg), where trace elements over adsorbed light gaseous hydrocarbons (${ \sum\nolimits _{i=2}^{5} C_i}$) anomalies showed good correlation with the existing oil and gas wells^25^.

Moreover, positive relationships between microbial population and hydrocarbon concentration in the near-surface sediments have been observed in various producing reservoirs worldwide^26^. The microbial studies can also help in identifying microseepages as the petroleum oxidizing bacterial colonies may develop in the near-surface due to the hydrocarbon gases seep^26^. The bacteria can consume hydrocarbon gases as their sole food and may appear in higher concentrations in the near-surface soils (or sediments). The higher populations of n-pentane, n-hexane oxidizing bacteria and methane oxidizing bacteria (MOB) show positive correlation with the abundances of gaseous hydrocarbons in the near-surface sediments, which can help to evaluate the prospects for hydrocarbon exploration^10,13,17,20^. A study from offshore West Africa found that, the hydrocarbon-oxidizing bacteria concentration ranges of 10^3^–10^6^ cfu/gm for the areas of hydrocarbon seepages, and the concentration was below 10^2^ for non producing areas. Generally, in oil and gas producing fields, bacterial growth has been found ranged between 10^3^ and 10^7^cfu/gm^27^. In producing oil and gas fields of Cambay basin (Kadi Kalol, Mehsana oil and gas fields), Jaisalmer Basin (Jaisalmer gas field) and Krishna Godavari basin (Ponnamanda and Tatipaka gas fields) in India, the hydrocarbon utilizing bacteria ranged between 10^6^–10^7^, 10^4^–10^5^, 10^5^ cfu/gm of soil. In the exploratory region of the Bikaner Nagaur basin, the bacterial growth of the sediments was reported as average 10^4^ per gram of soil. The compositional signatures of methane to ethane (C1/C2) and methane to propane ratios (C1/C3) can also be used as a proxy to determine the geochemical signature (oil, oil/gas, gas), where C1/C2 ratios less than 5 indicates oil bearing zones, between 5 and 18 indicates oil and gas condensate zones and between 18 and 50 indicates dry gas zones. For the corresponding zones the ratios for C1/C3 are given as 14, 33, and 80 respectively^28^. C1/C2 and C1/C3 ratios below 2 or above 200 indicate that the deposits are of no-commercial importance. We can determine the genetics of natural gas by analyzing the relative abundance of C1, C2, C3 i.e. Bernard ratio C1/(C2 + C3). The Bernard ratio of more than 500 indicates a biogenic origin of hydrocarbons, whereas as the ratio of less than 500 suggests a thermogenic origin^29^.

The molecular characteristics of gas may alter differently, due to different processes including post generation biodegradation or migration. Therefore, it is essential to find the origin of the gases. Carbon isotope study(^13^C/^12^C) is a widely used method to identify the origin of the hydrocarbon^30^.Previous studies have summarized different ranges for δ^13^C of CH_4_ values for natural gases^28–32^.

**Figure 1:**
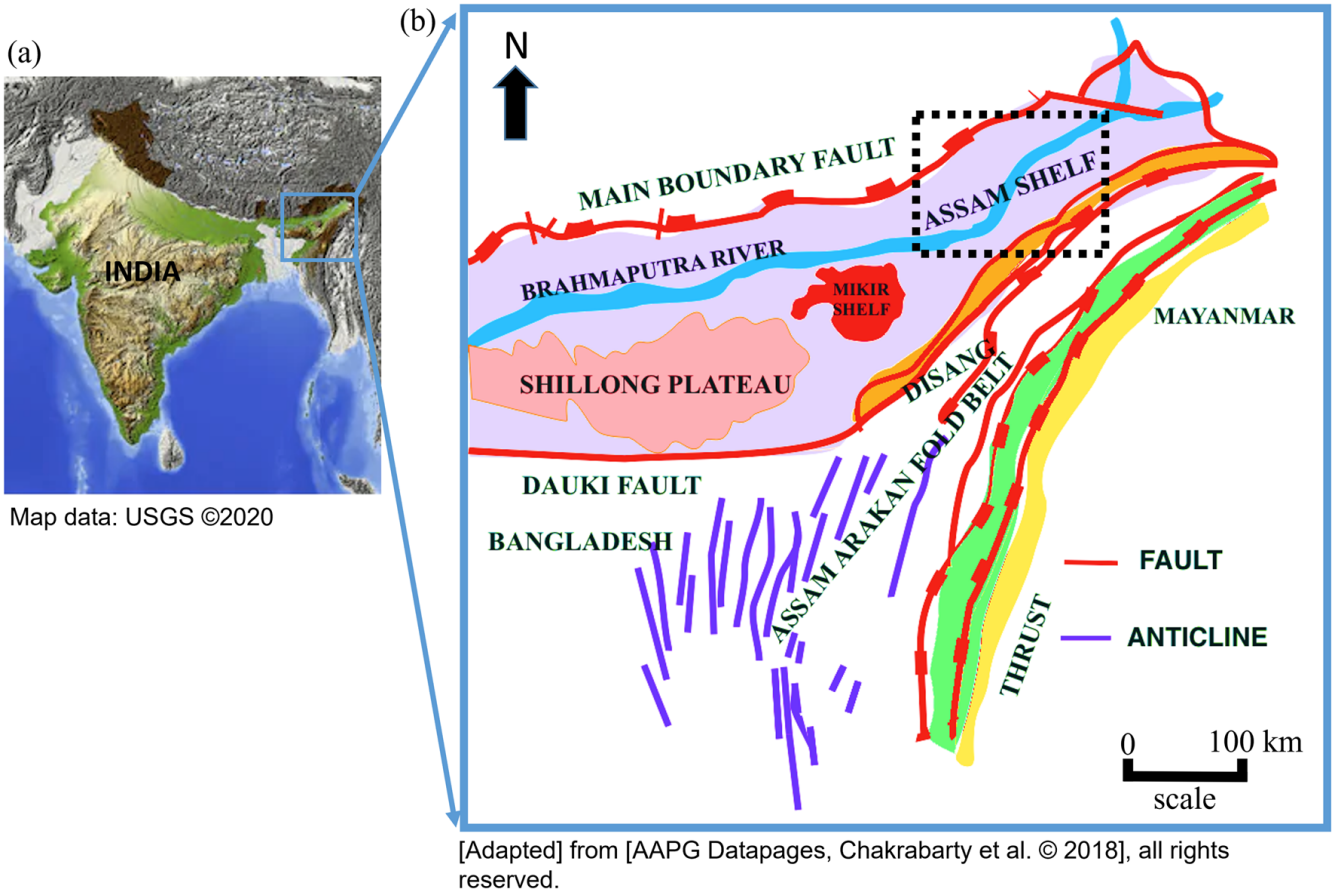
(**a**) Map of India^34^ (**b**) Geological Map of Assam Arakan Basin. The map shown in (**a**) is taken from USGS earth explorer. This is freely available at https://earthexplorer.usgs.gov. (**b**) is adapted from AAPG Datapages^35^.

**Figure 2:**
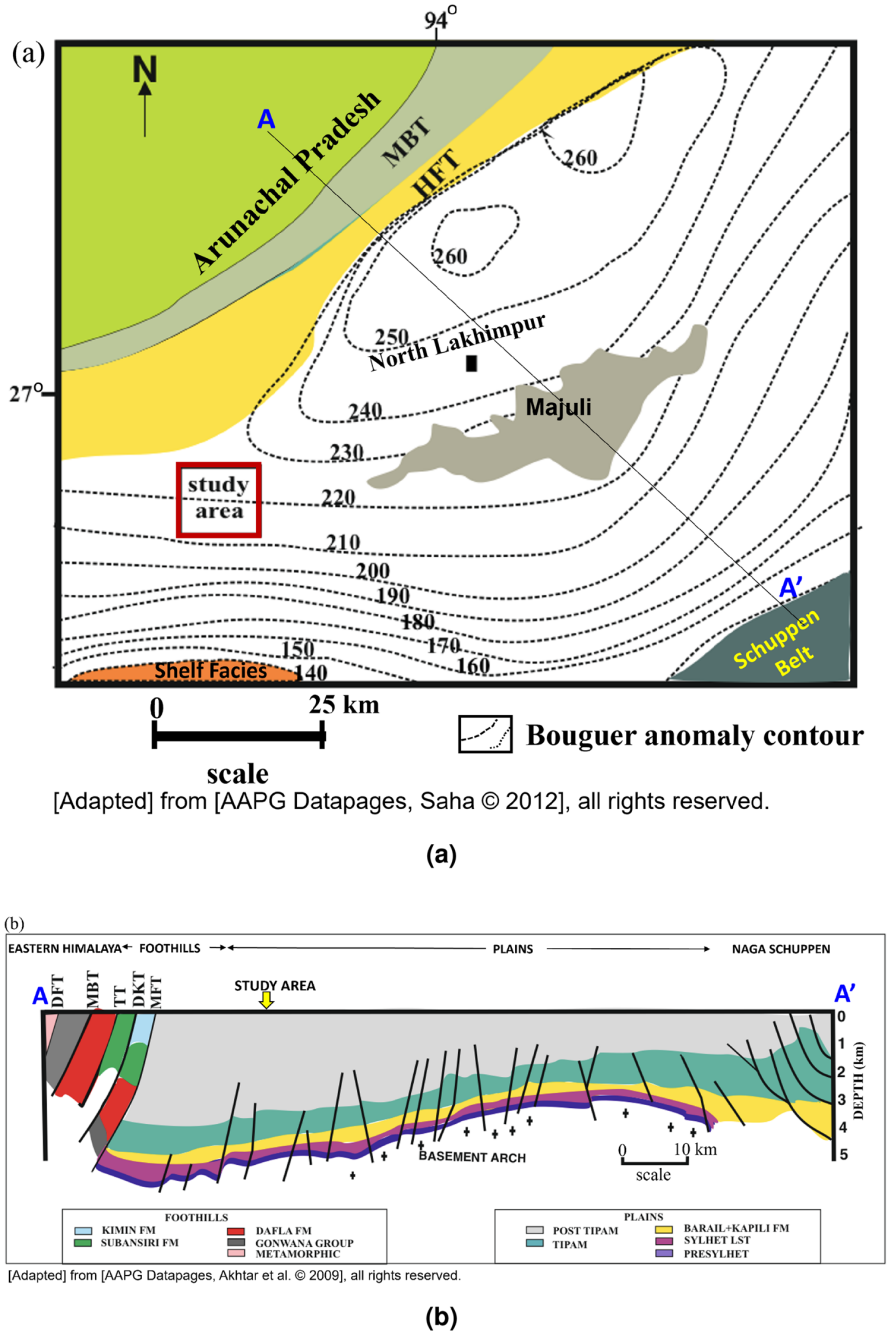
(**a**) Map showing structural setting of study area with gravity anomaly contour lines [*MBT* Main Boundary Thrust, *HFT* Himalayan Frontal Thrust]. The location of this map is marked by black dotted rectangle in Fig. 1. The figure is adopted from AAPG Datapages^39^, (**b**) Geological cross section extending North to south direction near to the study area (adopted from AAPG Datapages ©2009^40^).

Our study is based on macro seepage occurrences in Gohpur region, Biswanath (formerly known as Sonitpur) district in the northern part of Upper Assam, India, (Figs. 1,2). In spite of having visible macroseepages with possible hydrocarbon prospect, no exploration activities have been reported in the study area. However, at a 50 km of offset distance (in Lakhimpur district) seven exploratory wells were drilled between year 1995 to 2002. The exploratory data of those wells showed the presence of hydrocarbon in that areas. However, the exploration activities were stopped due to structural complexity, high depth of reservoir, etc. Also, a very limited number of the published research works on hydrocarbon prospect in the northern part of the Brahmaputra River are available^33^. With this motivation, we performed a detailed field investigation to collect appropriate samples for geochemical analyses near macro seepages. In this paper, first, we start by introducing the geological settings of the study area. Second, we describe the methodology of data collection, laboratory measurement including gas chromatography analysis, atomic adsorption analysis, carbon isotope study and cluster analysis. Finally, we present the interpretation of our analysis in the results and discussion section.

## Study area

**Figure 3:**
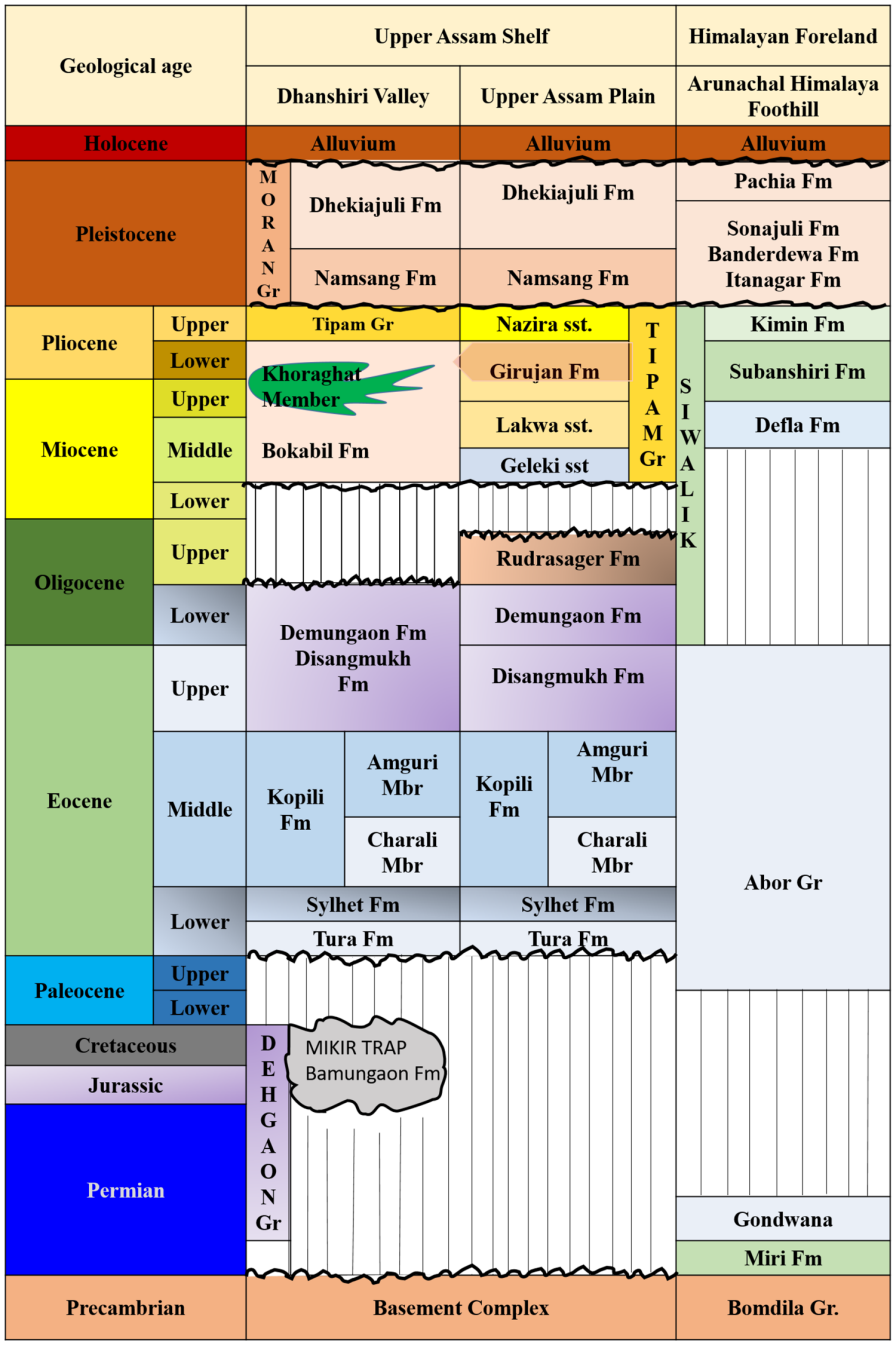
Stratigraphy of Assam Arakan Basin^41^.

**Figure 4:**
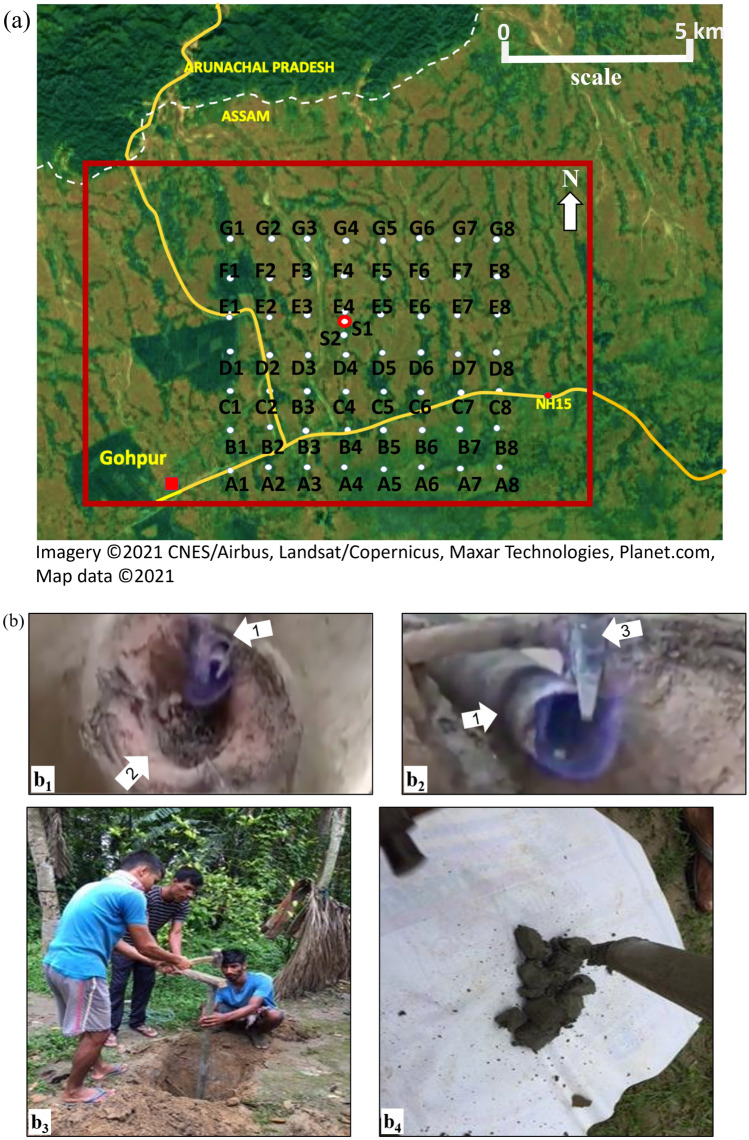
(**a**) Study area showing sampling locations^42^. The location of the macro seepage is indicated by the white dot with a solid red outline. The location of the red rectangle is shown in the Fig. 2 shows the same area as the red rectangle in this figure. Sample points locations are numbered as A1, A2,.. A8, B1, B2..,B8, and so on. The distance between A1 to A2 or A1 to B1 is approximately 1 km. There are two additional sample points S1 and S2 near the macroseepage location. (**b**) Photograph of Macroseepage location in Tingali Bori village (Photo $b_1$ and $b_2$); soil sample collecting from Kamalapathar (Photo $b_3$); soil sample collecting from Gopalpur village (Photo $b_4$).Notice the white arrows in photo $b_1$ and $b_2$; here arrow 1 shows the installed pipe for cooking purposes, arrow 2 shows the hole created as a stove to keep the cooking pot on, whereas arrow 3 shows a geological hammer for size comparison.The corresponding author captured the photographs shown in the Fig. 1b. Participants consent:The participants (Fig. 4b3) provided written informed consent for publishing their photograph in an online open-access publication.

The Assam Arakan Basin is one of the commercially oil and gas producing basins in India. The basin represents three major tectonic elements i.e., Assam Shelf, Naga Schuppen belt, and Assam-Arakan fold belt (Figs. 1 and 2). The basin represents a shelf-slope-basinal system, developed under the passive margin tectonic setting during Early Cretaceous to Oligocene. The tectonic history of the basin signifies different evolutionary trends including development of a number of horst and graben structures (Fig. 2b). A generalized startigraphic table is presented in Fig. 3. The basin covers more than 100 petroleum fields in the southern part of the Brahmaputra River, whereas the northern part of Brahmaputra River is poorly explored for hydrocarbon. Therefore, limited information is available on subsurface geology of the study area, except few scientific papers on exploration activities carried out in North Lakhimpur region by oil companies. The exploration block is at the distance of almost 50 km from the study area and their stratigraphy infers that Lakadong Member, Kopili Formation and Barail argillaceous unit are the source rock units with kerogen type II and type III^33^. Most of the source rock units of Eocene to Palaeocene age are in the thermally immature zone, where the potential source rocks were altered by diagenesis but not matured enough for the generation of hydrocarbon^36,37^. The study location is selected based on the active macro seepages around Gohpur, close to the Siwalik region (Fig. 4). The Siwalik region represents the low elevation mountain ranges formed in between the Lesser Himalaya (in the north) and the Brahmaputra Plains (in the south). The Siwalik sediments are exposed as a linear belt along the foot-hills in Arunachal Pradesh, where the Main Boundary Thrust fault (MBT)is overlying Gondwana formations in the north and the Himalayan Frontal Thrust Fault (HFT) is in the south trammeled from the underlying alluvium deposits. The Gondwana formations of the Permian age may also contribute as a matured source rock for the hydrocarbon generation. The equivalent Gondwana formations have significant hydrocarbon reserves in Dhansiri Valley, Borholla, Jamuguri and Dergaon Field in the Assam Arakan Basin. Moreover, equivalent formations in Damodar Valley Basins, Krishna Godavari Basin, etc., are considered as the most promising sources of unconventional shale gas resources^38^.

## Methodology

**Figure 5:**
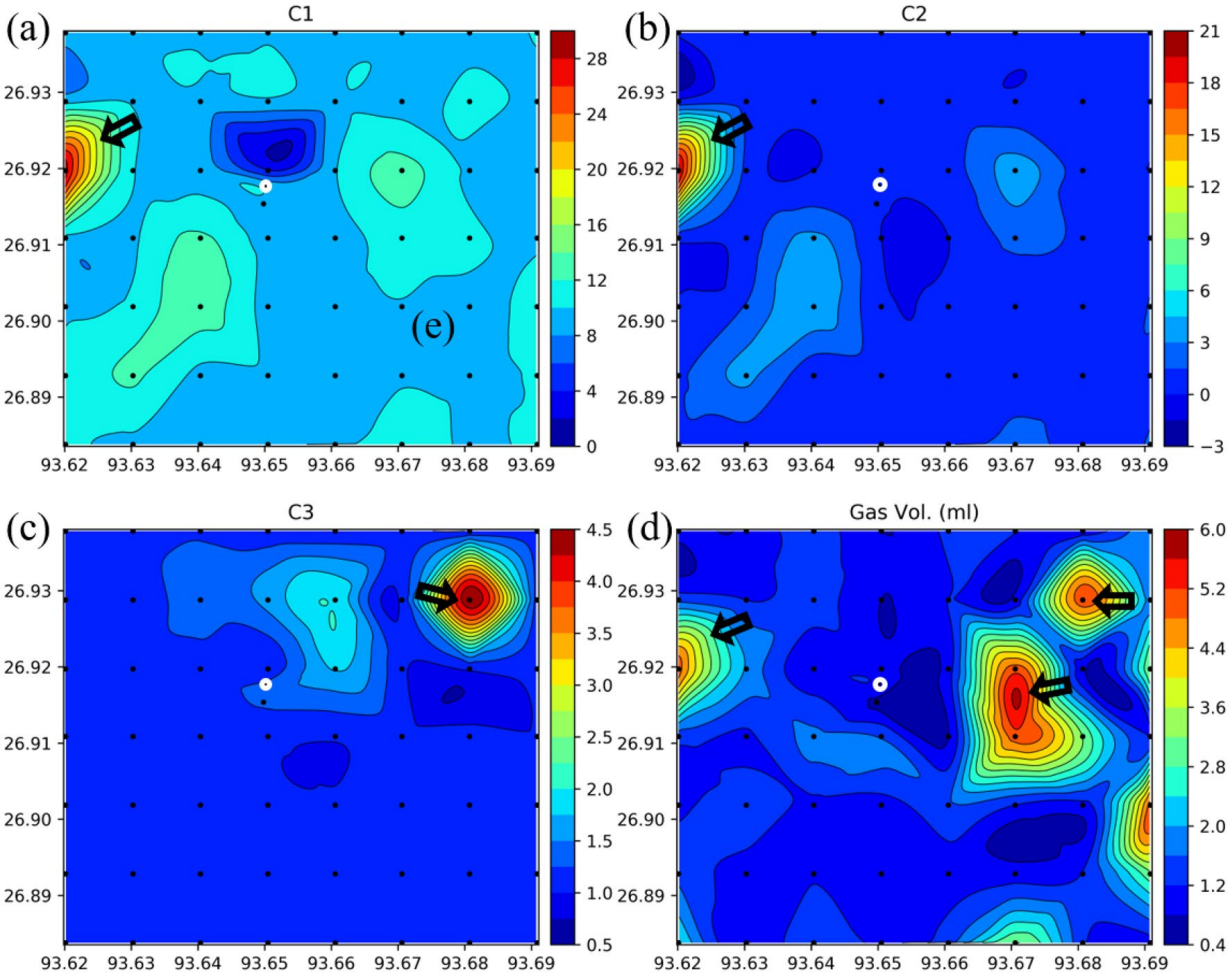
Gas chromatography measurements (**a**) C1, (**b**) C2, and (**c**) C3 concentration contour maps. Created the figures using using Python 3.5 Matplot lib^43^. The values of C1–C3 are in parts per billion (ppb). Contour maps of (**d**) free gas in soil. The colors and contours both represent the same parameter. X axis values longitude in degrees east whereas the Y axis latitude in degrees north.

**Figure 6:**
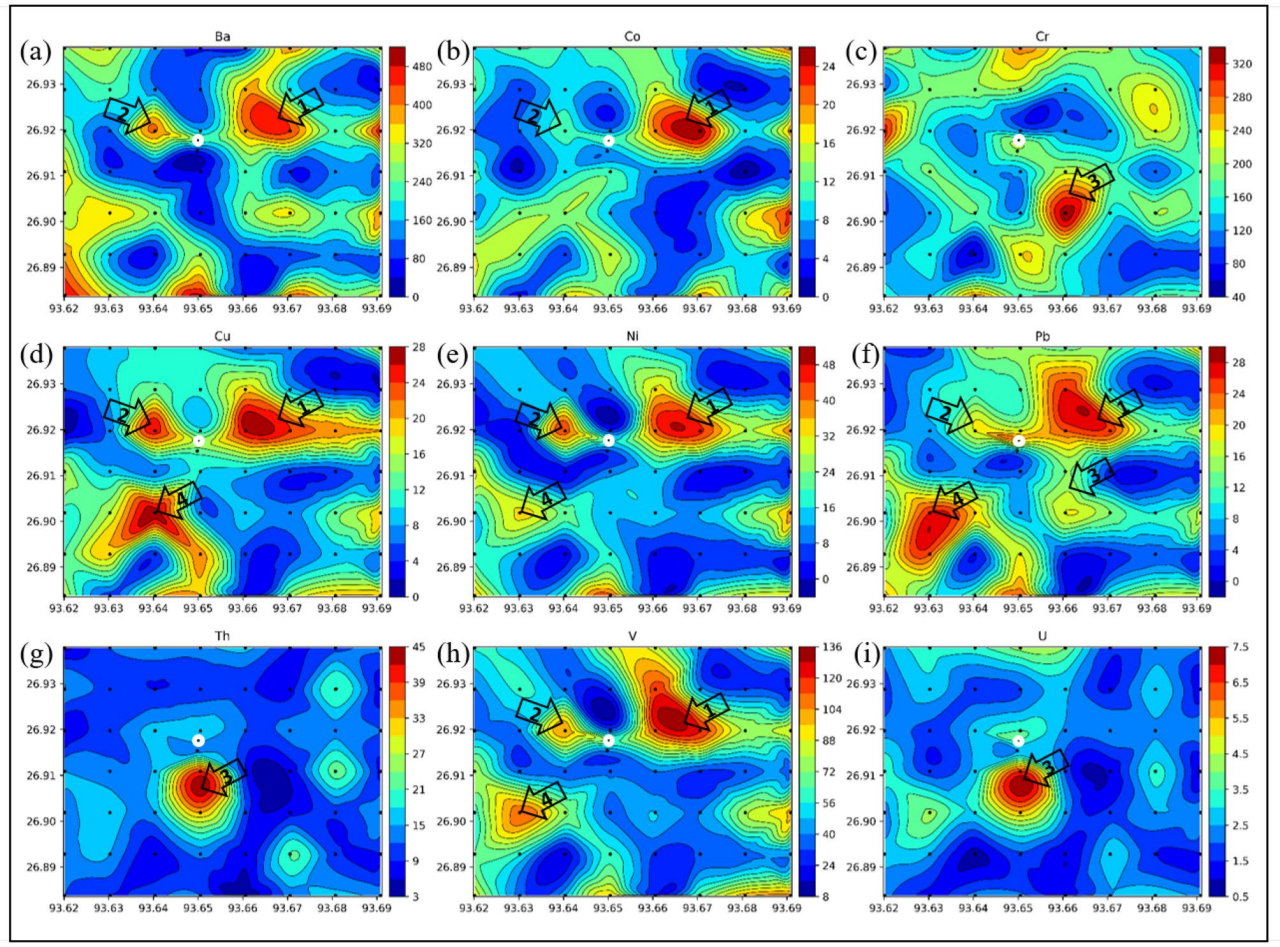
Contour maps showing trace element anomalies, (**a**) barium (Ba), (**b**) cobalt (Co), (**c**) chromium (Cr), (**d**) copper (Cu), (**e**) nickel (Ni), (**f**) lead (Pb), (**g**) thorium (Th), (**h**) vanadium (V), (**i**) uranium (U). The color shows the corresponding element concentration. All the element concentration values are in parts per million (ppm). Created the figures using using Python 3.5 Matplot lib^43^.

**Figure 7:**
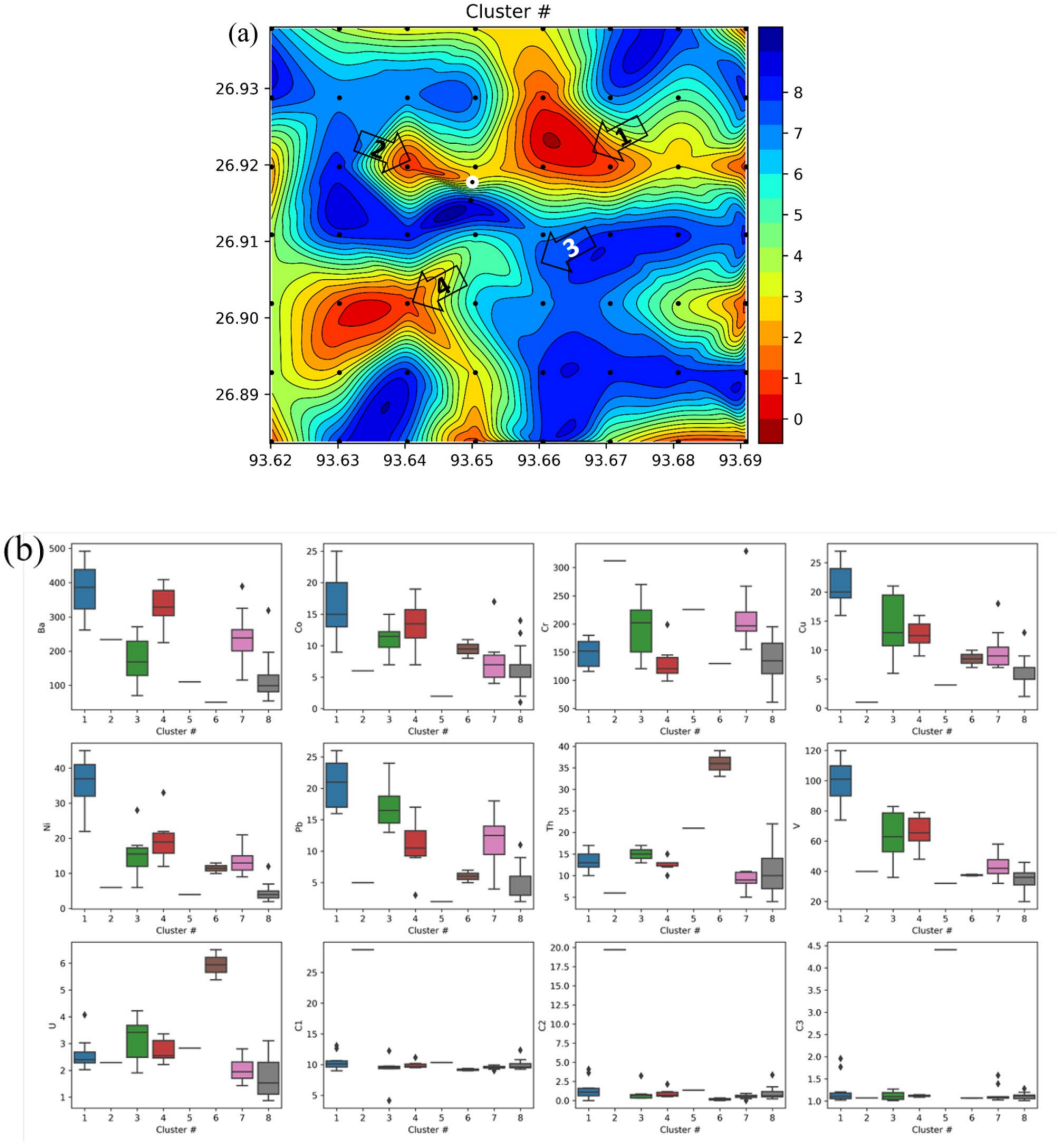
(**a**) Cluster map of trace elements. (**b**) Box plots of all the trace elements with respect to different cluster numbers. The cluster number 1 shows higher median values for most of the trace elements, similarly cluster number 8 shows the lowest values of median for all the trace elements^43^.

**Figure 8:**
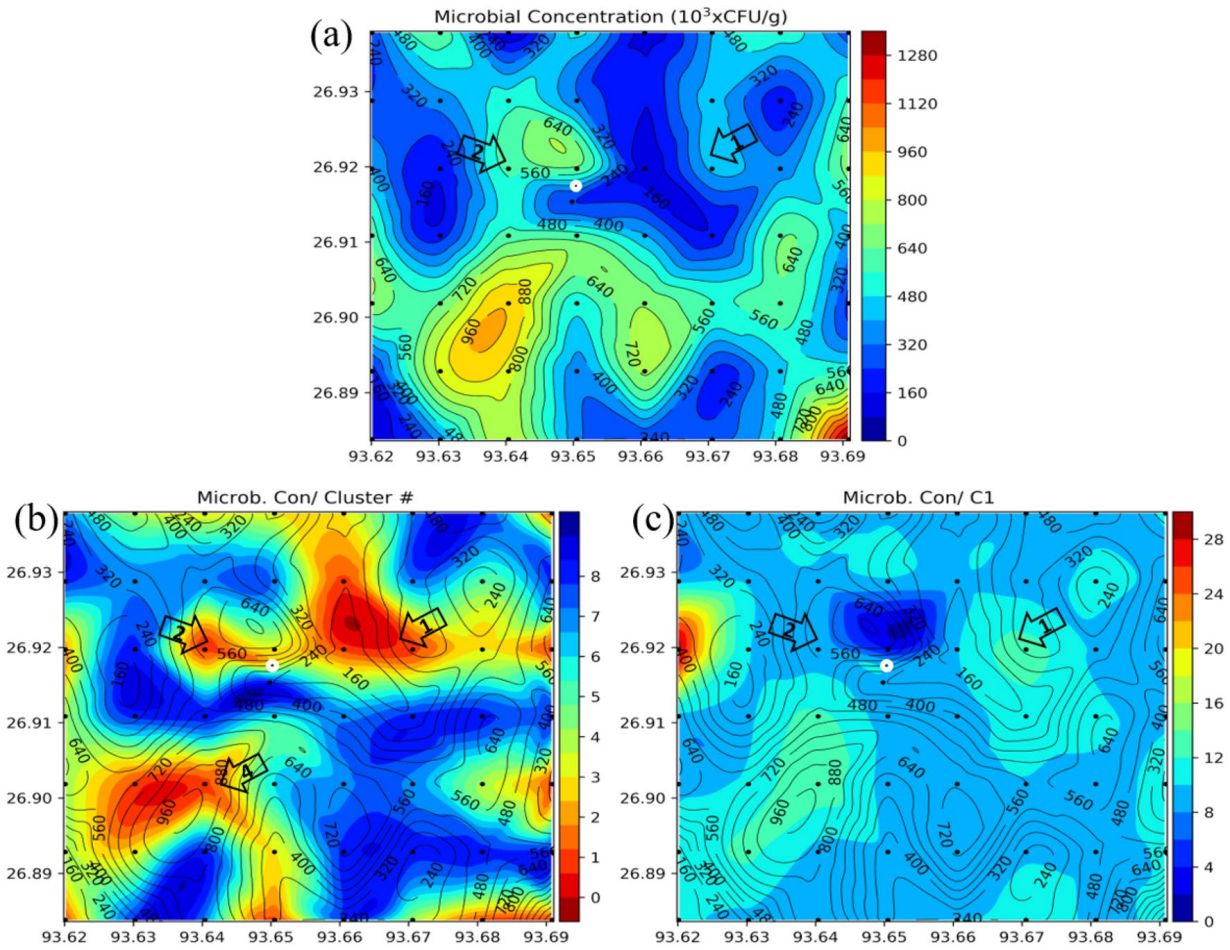
(**a**) Microbial concentration contour map; (**b**) integrated map of microbial concentration (on contours) and cluster number (on color); (**c**) integrated microbial concentration (on contours) and C1 (on color) map. We created the maps using using Python 3.5 Matplot lib^43^.

The methodology of the present study includes three parts: pre-field preparation, in-field sample collection, and laboratory analysis. We start pre-field preparation by determining the sample locations based on active macro seepages and then creating a topographic base map for field study with  1 km x 1 km grid cells (Fig. 4a). We add two additional points (S1 and S2) near the marco seepage area. The point highlighted as a red circle (sample location S1) on Fig. 4a is within 1 m distance of the two different marco-seepage locations. The total surveys area is 41.6 km^2^, with a total of 58 sample locations. We input the latitudes-longitudes of the sample locations in our GPS before the field visit. The GPS lead us to the sampling locations in the field. We drill and collect soil core samples from 2 to 2.5 m depth (Figs. 4b) for surface geochemical studies and from 0.5 to 1 m depth for microbial studies. Further, we perform an extensive laboratory analysis on the collected samples, including gas chromatography analysis, atomic adsorption spectroscopy, and microbial analysis (Figs. 5, 6, 7, 8).

### Gas chromatography analysis

We analyze the soil samples for light hydrocarbon gaseous contents like C1, C2, and C3 using gas chromatography (GC). We take 1 g of wet sieved 63 micron soil sample and treated with 40% orthophosphoric acid under partial vacuum in the presence of 20% KOH solution in the degasification unit. This whole process helps to desorb the hydrocarbon from the sample and the KOH absorbs the carbon dioxide gas released during the decomposition of carbonates. The desorbed gases are collected and 1 ml of gas sample is injected into the gas chromatography instrument equipped with the flame ionization detector. The GC instrument was calibrated using external standards with known concentrations of methane, ethane, and propane (Fig. 5).

### Atomic adsorption spectroscopy (AAS)

We digest the samples using PerkinElmer TITAN-MPS before the analysis using atomic adsorption spectroscopy (AAS). 0.3 g of powdered soil is used in Teflon vessels along with 6 ml of concentrated nitric acid (HNO_3_) + 1 ml of concentrated hydrogen fluoride (HF). Then, the treated samples are washed thoroughly with dilute nitric acid. We keep the samples in vessels airtight under 40 bar pressure and 175 °C temperature. Silica parts from the samples are removed and then 3% HNO_3_ is added to make up the total 25 ml solution. We measured the concentration of trace metals Ba, Co, Cr, Cu, Ni, Pb, Th, U, and V (Fig. 6).

### Cluster analysis

We measured the concentration of, nine different elements in AAS study and three different gases. We want to identify higher concentration zones of all these elements, but analyzing twelve different maps at the same time is challenging. So, we use an affinity propagation (AP) clustering technique to divide the data into eight different clusters. We identify the cluster with the highest value of the total normalized concentration of all the twelve parameters, and index this cluster as cluster #1. Similarly, we index the other clusters (2, 3, ...) in decreasing order of total concentration (Fig. 7).

AP clustering is an unsupervised clustering method, unlike k-means clustering, which does not require the number of clusters as an input. AP is applicable to small-size data sets. AP clustering algorithm forms clusters by sending messages between data points. AP is a graph based clustering method. In this method, Euclidean distance-based similarity between all the pair of data points is computed. The idea is to put higher similarity points in one cluster. AP maximizes the total similarity between exemplars and related data points to generate clusters. An exemplar is a representative example of a cluster, and a member of the input set^42–46^.

### Microbial analysis

The soil samples of about 100 gm are collected from a depth of about 0.5 to 1 m in pre-sterilized whirl-pack bags under aseptic conditions. As soon as the samples are brought to the surface, they are stored at 2 to 4 °C and stored in cryogenic conditions. Isolation and enumeration of methane-oxidizing bacteria and ethane/propane (C2/C3) oxidizing bacteria for each sample is carried out by standard plate count (SPC) method^8^. We created microbial concentration contour maps and identified the anomalous zones (Fig. 8).

### Carbon isotope analysis

We analyzed a total 8 samples using a gas chromatography isotope ratio mass spectrometry (GC-C-IRMS). The isotope analytical system includes the GC, the isotope ratio mass spectrometer (IRMS), and the interface component. We did combustion of organic carbon and converted into CO_2_, then we measured the isotopic masses of CO_2_. The IRMS allows the precise measurement of stable isotopes. Here, the carbon isotopic composition is in per mil ($\%\circ$) relative to the Pee Dee Belemnite (PDB) with the precision of ±0.5$\%\circ$. We referred to the internationally accepted standard Pee Dee Belemnite (PDB), a calcium carbonate fossil of Belemnitella americana from Cretaceous Pee Dee formation in South Carolina, and this is assigned a ^13^C value of 0$\%\circ$^31^. The isotopic properties for wet thermogenic gas range from − 25$\%\circ$ to − 40$\%\circ$ (PDB), dry thermogenic gas range from − 15$\%\circ$ to − 40$\%\circ$ PDB. The isotopic properties of bacterial gas range from − 60$\%\circ$ to − 110$\%\circ$ (PDB)^36^. ^13^C of methane and C1/(C2 + C3) molecular concentration of the gas on the Bernard plot help to determine whether the gases are mixtures of bacterial and/or thermogenic. The relationship between ^13^C of methane and ^13^ of ethane is also useful to define the mixed gas properties^30^.We created a modified cross plot diagram and identified the origin of the gas based on the carbon isotope ranges and C1/C2 + C3 ratio. We used a summarized range of ^13^C of methane from the previous literatures^28–32^.

## Result

### Adsorbed gas and trace element concentration

The total adsorbed gas volume ranges from 0.8 to 5 ml and the significantly higher concentration anomaly zones are present towards the eastern part of the study area (Fig. 5). The highest gas volume is observed at sample points E1, E6, D6, F7, D7, C7, A6, and A1, creating a radial pattern around the active macro seepage zone near E4. The data indicates that the total gas volume is significantly low at active macro seepage (1.1 ml at location no. S1) and nearby areas (0.94 ml at D1 and 1.22 ml at E5). Methane and ethane concentration are low near active seeps (Fig. 5a and b). Propane concentration is higher at sample point F7 and nearby areas (Fig. 5c). Low concentration of adsorbed gas volume at the active macro seepage location (sample point S1) and nearby area may be due to long-term leakage of hydrocarbons as macro seepage which favors the development of a diverse array of chemical and mineralogical changes in the soil, leads to a high amount of free gas compared to adsorb gas. This may be due to gas dispersion from active macro seepage associated with high temperatures and pressures.

The concentration of trace element varied in wide ranges for Ba (54–492 ppm), Cu (1–25 ppm), Cr (61–329 ppm), Ni (1–42 ppm), Pb (2–48 ppm), Th (2–32 ppm), U (4–39 ppm), V (19–133 ppm) and U (0.87–6.5 ppm) with an average mean concentration value of 240 ppm, 10 ppm, 164 ppm, 12 ppm, 17 ppm, 13 ppm, 13 ppm, 60 ppm and 2 ppm respectively. It is observed that the concentration of trace elements is significantly higher than their average concentration at different sampling points. In our study, we identified four zones with high anomaly concentrations of trace elements (Fig. 6). The trace element Ba concentration (Fig. 6a)shows two high concentration anomaly areas towards the northeast part of the area, marked in the map with arrow (1 and 2). The higher concentration anomalous zone shown by the trace elements like Co, Cu, Ni, Pb and V are shown as zone 1 in Fig. 6b,d–f,h. Another high concentration anomaly zone (2) is identified towards the northwest side of the active macro seepage location. The trace elements Cr, Th, U are showing high concentration anomaly towards the south of the active seepage location, marked as at zone 4 (Fig. 6c,g,i). Cluster map of trace elements (Fig. 7a) also signifies 3 prominent zones of high trace element concentration. In the box plots of the trace elements (Fig. 7b), the cluster number 1 shows higher median values for most of the trace elements, while cluster number 8 shows the lowest values of median for all the trace elements.

### Microbial analysis

In our study area, the hydrocarbon-oxidizing bacteria count ranges from nil to 1.37 × 10^6^ cfu/gm. Most of the soil samples show greater than 10^4^ cfu/gm of soil of bacterial growth, which confirms the seepage of lighter hydrocarbon occurrences from the subsurface oil and gas deposits (Fig. 8a). The anomaly distribution map of POB population shows distinct microbial anomalies in zone 4 and nearby area. Figure 8b shows comparatively low population of hydrocarbon oxidizing bacteria towards zone 1 where the trace element contractions are significantly high. Figure 8c exhibits the existence of high densities of bacteria in black-circled locations in the sampling area, which confirms the high probability of detecting hydrocarbon in the SW part of the sampling area (zone 4).

Integrated map of cluster number and microbial concentration show a high trace concentration in zones 2 and 4 (Fig. 8b). Low concentration of C1 just above the microbial anomaly signifies the degradation phase of hydrocarbon due to consumption by the bacteria in that zone, whereas anomalous high concentration of microbes in the adjacent areas indicate the presence of microseepages in the near-surface sediments.Thus, the anomalies observed can be the indicators for occurrences of light hydrocarbon seepages from the subsurface petroleum deposits.

### Genetic origin of the gases

**Figure 9:**
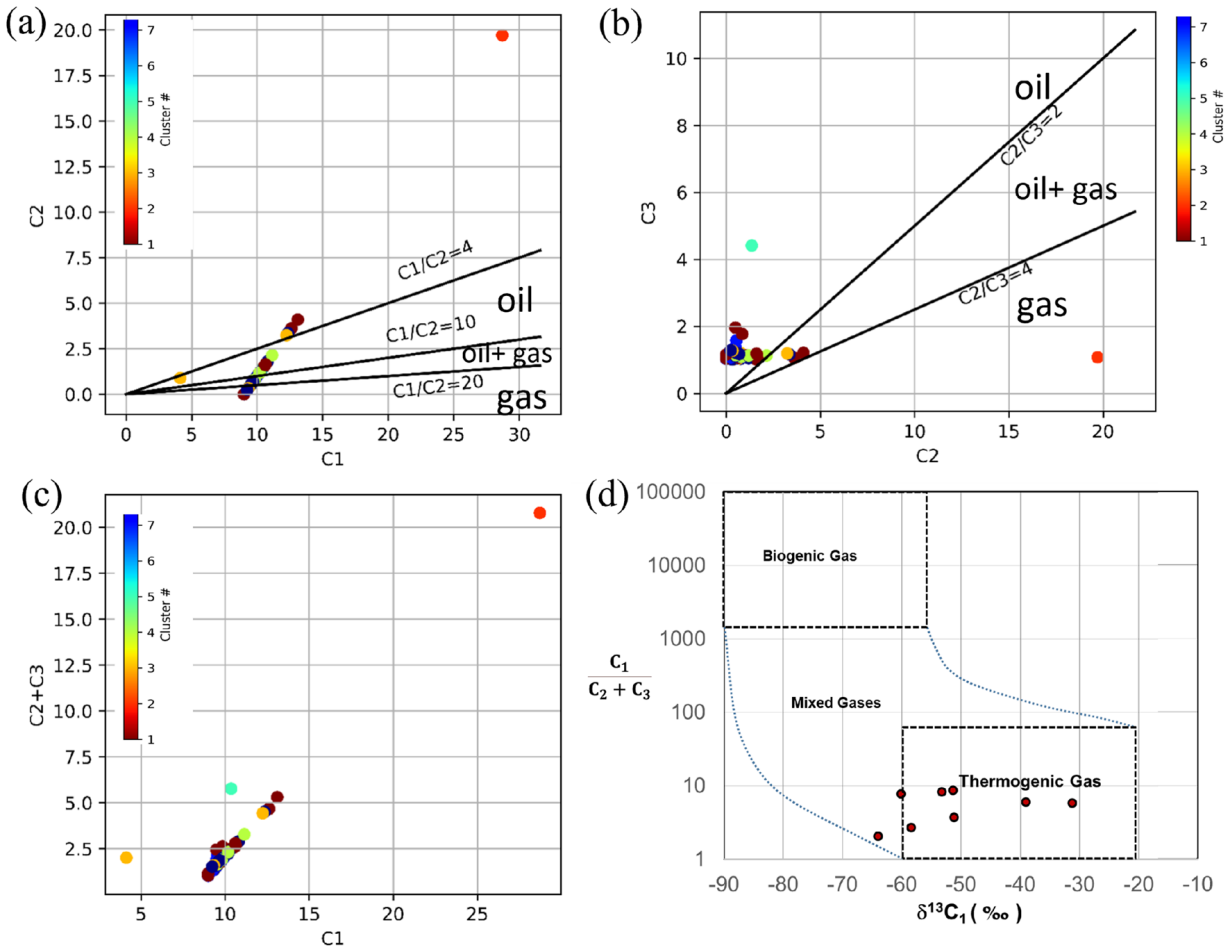
Scatter plots of (**a**) C1 vs C2 + C3, (**b**) C2 vs C3, (**c**) C1 vs C2 + C3 plot. The points are colored by cluster number^43^. (**d**) Cross plot of the parameters C1/(C2 + C3) versus 13C-CH4. Two mixing trends (dashed lines) of thermogenic with bogenic gases (modified diagram^30^).

Most of the data points (93% of sample population) on the the scatter plots of C1 vs C2 (in Fig. 9a) follow a linear trend, which suggests that most of the hydrocarbons have the same origin (either biogenic or thermogenic). A high correlation of C1 with C2 + C3 indicates that hydrocarbon in most of the samples have been derived from thermogenic source (Fig. 9b). Also, the isotopic results (in Fig. 9d) suggest the presence of thermogenic and mixed biogenic-thermogenic gases. Additionally, C1/C2 vs C2 + C3 cross plot indicate that the presence of oil, oil and gas condensate (Fig. 9c).

### Discussion

High concentration of trace elements in the sediments can be due to the interaction of hydrocarbon gases with clays. The clays can hold the hydrocarbon gases in three forms, i.e. adsorbed, free and dissolved with water. The hydrocarbon gas molecules can create organic film around the clay molecule surface or it can attach to the clay lattice. Both of these processes affect the ion exchange capacity of clays, which will result in a reducing condition. On the other hand, when no hydrocarbon gases are present in the clays, it will cause an oxidizing condition. In reducing conditions, the clays attract trace elements to attach to its lattice system and the trace metals form organometallic complexes with clay particles, whereas in oxidizing conditions there are no formation of organometallic complexes^45–49^. Such that, a boundary develops between the reducing and oxidizing zones by the trace elements. In our study area we observed a halo pattern developed by the high concentration of trace elements encircling the active hydrocarbon seepage (Fig. 7a), which signifies the presence of hydrocarbon gases in the sediments. We suspect that the hydrocarbon gases seep from a subsurface hydrocarbon accumulation. The compositional ratio of C1/C2 + C3 and 13C value of methane Fig. 9d) suggests that the hydrocarbons are mostly thermogenic, along with small proportions of mixed biogenic-thermogenic gases.The biogenically produced methane mixes with the thermally generated gases, which is seeping to the surface as macro and micro seeps. Variation of isotopic composition is due to the mixing of both the thermogenic and biogenic gases.

**Figure 10:**
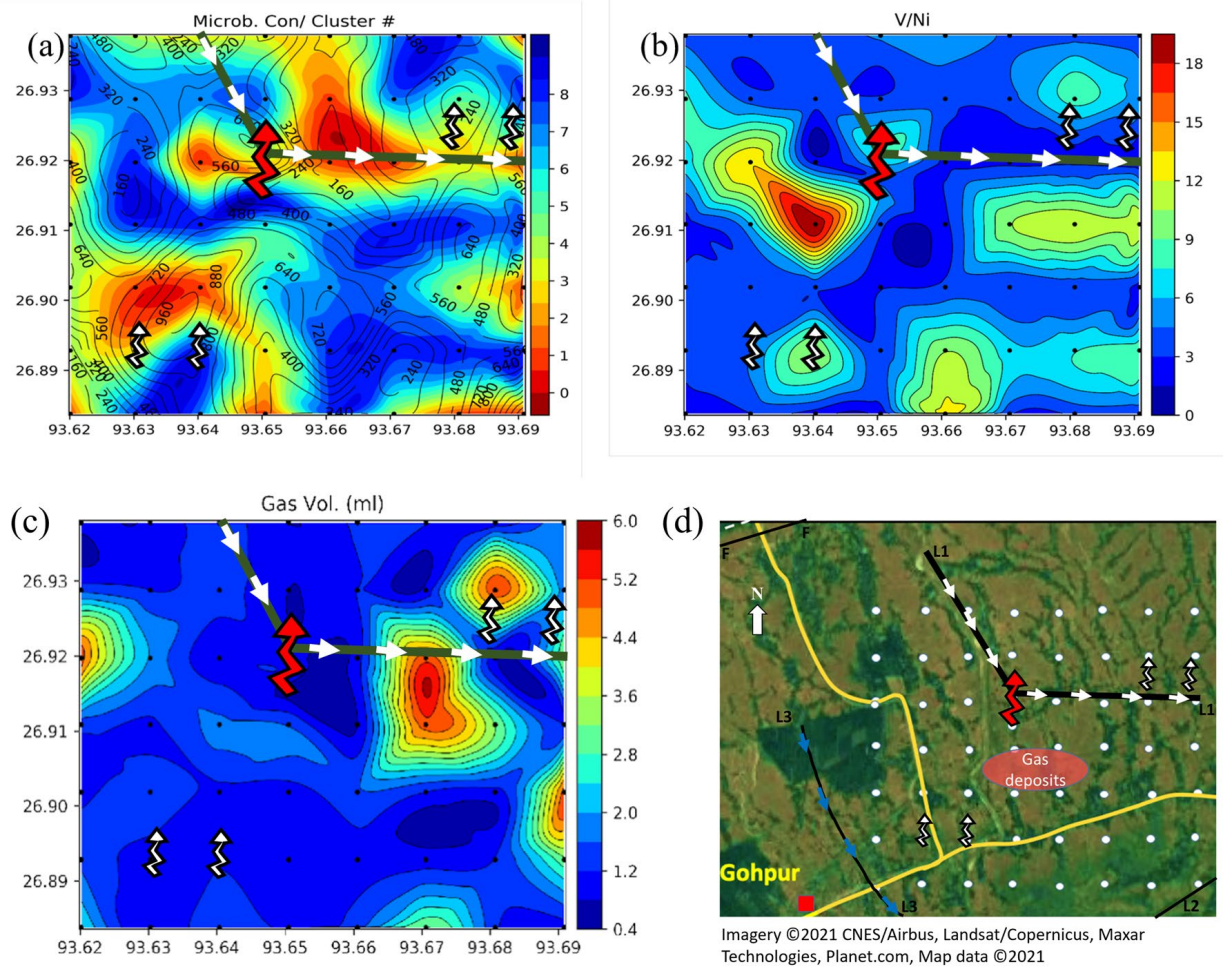
The gas migration routes (in white arrows) on top of the structural lineament L1 (solid green line) along with the active macro seepage (zigzag red arrow) and interpreted micro-seepage (zigzag white arrow) displayed on microbial concentration contour map (**a**) Cluster map of microbial concentration^43^, (**b**) V/Ni ratio contour map^43^, (**c**) gas volume contour map, (**d**) Gas migration pathways along the lineaments^42^.

Moreover, most trace elements especially V, followed by Co, Cr, Cu, Ni, Pb, U and Th, show correlation with microbial concentration, which is closely related to bacterial anomalies that feed on the seeping hydrocarbons. We identified four zones (1, 2, 3, and 4) of interest based on the elemental concentrations and microbial analysis. A high concentration of uranium in zone 3 along with a high microbial concentration. Uranium has two valence states, i.e. oxidized form, the uranyl ion (U02++) and non-oxidized uraninite (U02), where uranium tends to migrate from an oxidizing (U02++) to a reducing (U02) environment. The higher concentrations of uranium in the study area may be the indication of a reducing condition^50,51^. V/Ni ratio shows a gradually decreasing trend from NNW to S and SSE direction, the highest value of V/Ni ratio 19 ppm occurs at the sample point G3. We determine the hydrocarbon migration pathway based on the microbial concentration cluster map (Fig. 10a)and high to low V/Ni ratio (Fig. 10b), we indicate it as L1, L2 and L3 lineaments (Fig. 10c). V/Ni ratio decreases through F3 (13.3), A4 (12.5), C7 (11.7), E2 (11.7), A4 (10.3) in ppm (Fig. 10b). Apart from observing a change in V/Ni ratios, we also found higher concentrations of some trace elements (Ba, Cu, Co, Ni, V, Pb), propane (C3) and total adsorbed gas volume on both sides of the lineament L1. Cluster map based on trace elements and gas concentrations indicates that zone 1 follows almost the same trend with the lineament (L1) (Fig. 7). Further geological investigation indicates the presence of is a north to north-west trending fault known as Himalayan Frontal (HFF), which is parallel to L2, at less than 30 km from the center of the study. In the Siwalik region, where the HFF occurs, there are many other lineaments, which have played a significant role in providing the macro and micro seepage routes^3^. We believe the seeped gases charge the sediments present on both sides of the lineament; hence, most trace elements show a high concentration anomaly. We suspect that these lineaments (L1, L2, and L3) are faults or fractures and allow for a long distance tertiary lateral migration of hydrocarbon causing the macroseepages (Fig. 10d). Alternatively, the micro seepages are most likely caused by the vertical migration through (sediment or rock) pores from deeper hydrocarbon resources or shallow biogenic hydrocarbon accumulations.

**Figure 11:**
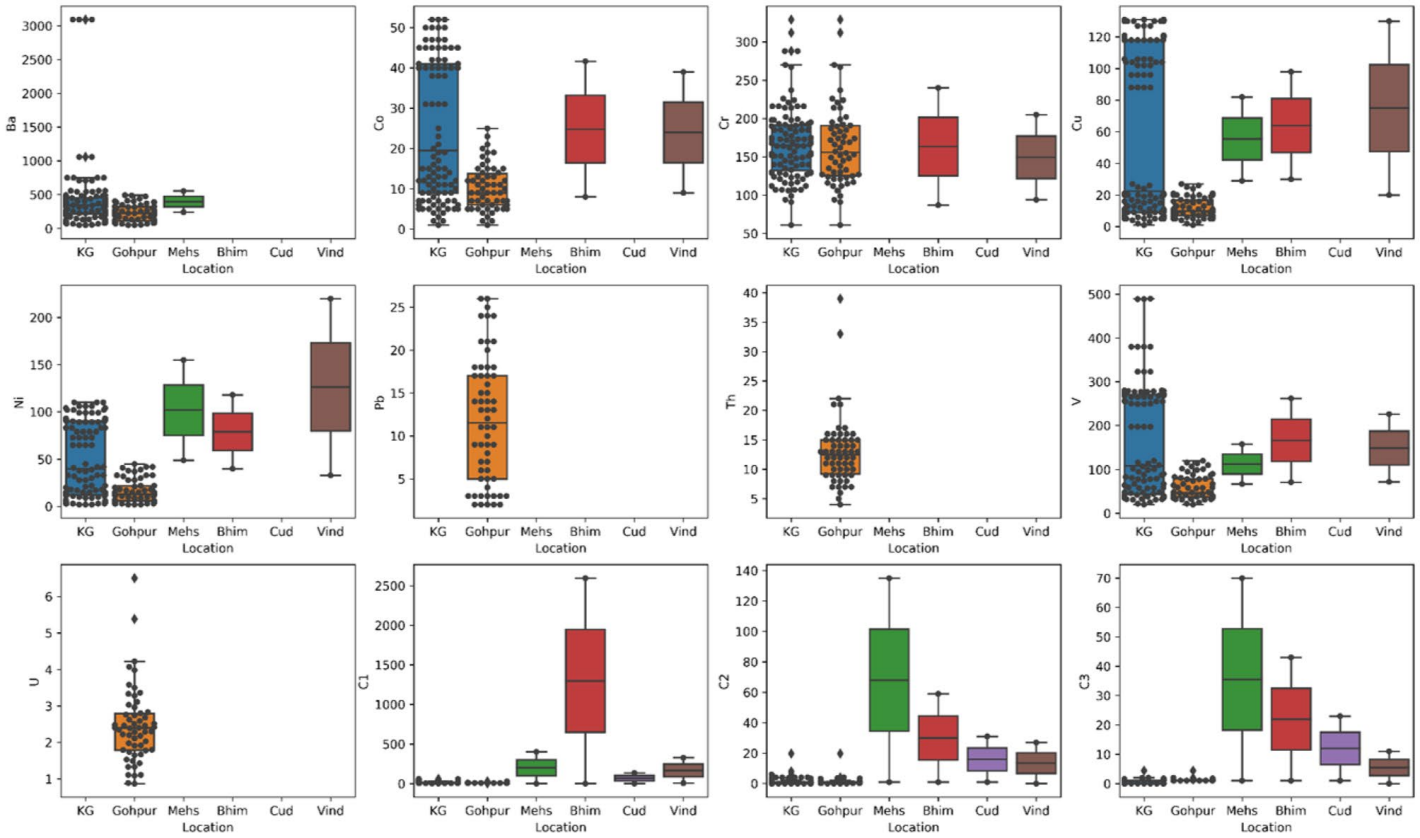
A comparison of trace element ranges of study area with some hydrocarbon producing and non producing areas. [*KG* Krishna Godavari Basin,* Mehs* Mehsana area in Cambay Basin, *Bhim* Bhima Basin, *Cud* Cuddapah Basin, *Vind* Vindhyan Basin]. The black dots show element concentration values on different samples. The black box shows data range between 25 to 75 percentile of the data, whereas the whiskers represent 5 to 95 data percentiles, the line in the middle of the black box represents the median value^43^.

We also compared the trace element ranges of the study area with some hydrocarbon producing and non-producing fields from different Indian basins to better understand the hydrocarbon potential of the study area. We used published data on adsorb gas content, trace element, and microbial concentrations from parts of Krishna Godavari Basin, Cambay Basin, Bhima Basin, Cuddapah Basin, and Vindhyan Basin of India (Fig. 11). We found that most of the trace element concentrations of the study area fall in the same range as the producing fields of Krishna Godavari Basin and Cambay Basin.

Although the analyzed data cannot reveal the amount of subsurface hydrocarbon accumulation or its depth. Also, the data cannot accurately predict economic success or failure of exploration in this area. Based on the structural features and our analysis in this paper, we believe that the long-distance lateral migration occurs through thrust faults from the organic rich Gondwana shales of the Himalayan thrust zone^3^. Our methodology could further integrate with other geo-scientific studies (i.e., seismic data), which may evolve with the holistic picture of identification of hydrocarbon bearing formations within this frontier area of NE India.

## Conclusion

There are visible gas seepages in the study area, which indicate the presence of subsurface gas accumulations. The trace element concentration, adsorbed gas, and microbial analysis also suggest that the study area has experienced long-term macro and micro seepages. Interaction of seeped gases with the sediments leads to the higher concentration of trace elements. Gas chromatography and carbon isotope analysis on samples indicate that the gases contain primarily methane with a small quantity of ethane and propane. The presence of methane as a major proportion in the gases suggests the bacterial action in the sediments, which gets mixed with thermally occurring seeping gases. The isotopic values indicate that the gases are mostly of thermogenic origin. The geology of the study area controls the long-distance lateral migration of the light hydrocarbon gases. Anomalously high concentrations of trace elements and microbial concentrations are located above outcropping lineaments. So, we believe that the gaseous hydrocarbon migration to the surface is channelized by major faults and fractures. Cluster analysis is a useful tool for recognizing potential areas, which coincides with the known petroleum deposits. The study area shows a probable hydrocarbon prospect; for future exploration we recommend geophysical data acquisition including 2D, 3D seismic and exploratory well logging.
